# Investigation of the Second Harmonic Generation at the Water–Vacuum Interface by Using Multi‐Scale Modeling Methods

**DOI:** 10.1002/open.202200045

**Published:** 2022-08-11

**Authors:** Tárcius N. Ramos, Benoît Champagne

**Affiliations:** ^1^ Laboratory of Theoretical Chemistry Namur Institute of Structured Matter (NISM) University of Namur Rue de Bruxelles, 61 B-5000 Namur Belgium; ^2^ Laboratory of Theoretical Chemistry Namur Institute of Structured Matter (NISM) University of Namur Rue de Bruxelles, 61 B-5000 Namur Belgium

**Keywords:** air–water interface, embedding effects, first hyperpolarizability, second harmonic generation, sequential MD-then-QM approach

## Abstract

The Sequential Quantum Mechanics/Molecular Mechanics scheme has been enacted to perform a systematic investigation of the polarizability (*α*) and first hyperpolarizability (*β*) responses at the water–vacuum interface. After performing classical molecular dynamics simulations to provide snapshots of the structures, quantum chemistry calculations of the linear and nonlinear optical responses have been performed for clusters of five water molecules at the time‐dependent DFT level in combination with different embedding schemes, ranging from point charges to polarizable point charges, with and without local field effects. When going from the bulk to the interface, the main observations of these calculations encompass i) a modest increase of the average polarizability but an increase by about a factor of two of its anisotropy, ii) an increase by about 20 % of the *β_HRS_* response, accompanied by a small increase of its depolarization ratio, and iii) a net increase of the component of the *β* tensor normal to the interface (*β_zzz_*) as well as of *β*_*//*_. Globally, the interfacial effects on *β* are localized at the first molecular layer while they are observed up to the fourth molecular layer on *α*.

## Graphical abstract

**Combining classical molecular dynamics with DFT** quantum chemistry calculations and different embedding models, the polarizability *α* and the first hyperpolarizability *β* of small water clusters at the water–vacuum interface have been evaluated, highlighting that the interfacial effects on *β* are localized at the first molecular layer while they are observed up to the fourth molecular layer on *α*.

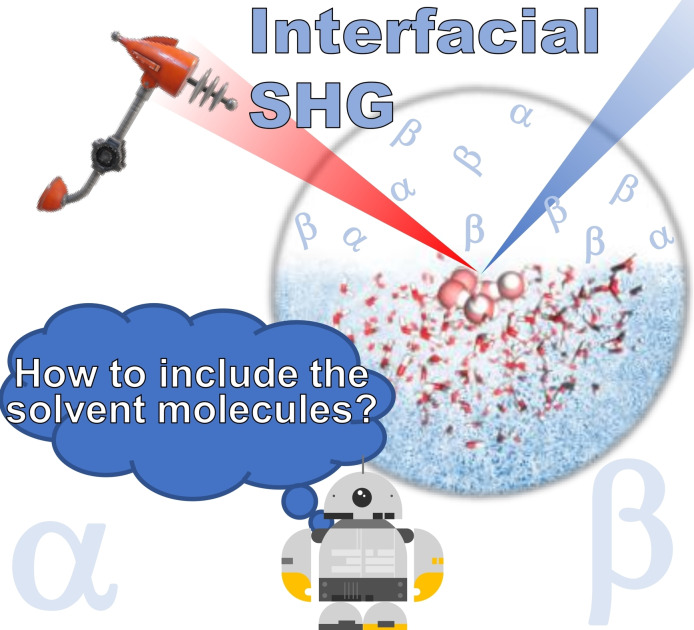

## Introduction

Nonlinear optical (NLO) spectroscopies are efficient techniques to characterize interfaces.[^1^, ^2^, ^3^, ^4^] The latter are non‐centrosymmetric and therefore they exhibit second‐order NLO responses, characterized by the first hyperpolarizability (*β*) at the molecular scale and by the second‐order NLO susceptibility ($\chi^{\left(2\right)}$ ) at the macroscopic scale. Among these phenomena, Second Harmonic Generation (SHG) is associated with the conversion of pairs of photons of energy ℏω into photons of energy 2ℏω . Another phenomenon is Sum Frequency Generation (SFG), which describes the conversion of photons of energies $\hbar \omega_{1}$ and $\hbar \omega_{2}$ into photons of energy $\hbar \left(\omega_{1}+\omega_{2}\right)$ .^[5]^ Both SHG and SFG phenomena have been used to probe the structure and dynamics of interfaces, be there in materials or life sciences.[^6^, ^7^, ^8^, ^9^] SHG is also employed to determine the first hyperpolarizabilities of molecules in solution, either through its coherent response (electric‐field induced SHG ‐ EFISHG)[^10^, ^11^] or incoherent one (hyper‐Rayleigh scattering).[^12^, ^13^, ^14^, ^15^, ^16^]

Among interfaces, the water–vacuum interface has been the target of many studies in different areas, including atmospheric chemistry, biological, organic chemistry, and others, see Refs. [17, 18] and the references cited therein. For instance, it has been shown that reactions at the interfacial water region occur faster than in the bulk, even though the reasons remain unclear. Improved understanding and characterization of the interfacial region call for joint experimental and theoretical simulation studies, including by using the interface‐sensitive SHG technique.

From a theoretical/quantum chemistry (QC) point of view, to support and help interpret the experimental characterizations, the interfacial SHG responses have been calculated using several computational schemes. A straightforward approach consists in calculating $\chi^{\left(2\right)}$ as the tensor sum of the molecular *β* responses, of which the molecular structures consist of snapshots extracted from (classical) Molecular Dynamics (MD) simulations. For instance, this methodology has been employed for describing the second‐order NLO responses of chromophores in biological lipid bilayers[^19^, ^20^] (of which each leaflet behaves like an interface) and functionalized surfaces.[^21^, ^22^]

Recently, the $\chi^{\left(2\right)}\left(-2\omega ;\omega ,\omega \right)$ response of the water–vacuum interface has been calculated as the product of two quantities, i) an orientational average parameter (< OR >) of the water molecules, evaluated from the snapshots of the MD simulations, and ii) a unique, parameterized, *β* value of the water molecule, so that $\chi^{\left(2\right)}$ ∼ < OR >*β*.^[23]^ However, that methodology neglects the effects of the surrounding molecules on the individual *β* responses. Lately, the effects of the surrounding molecules have been accounted for by calculating the *β* responses of each water molecule in their electrostatic embedding, so that the *β* values differ from one molecule to the other. Then, in that study, considering the MD snapshots, average *β* values have been monitored as a function of the interface distance.^[24]^ From this methodology, $\chi^{\left(2\right)}$ is estimated as the < OR *β* > average instead of the < OR >*β* product. Yet, these two approaches have resulted in different descriptions of the $\chi^{\left(2\right)}$ responses, which question the currently assumed approximations. An alternative methodology to describe the SHG responses avoids defining a molecular response (i. e., *β* of the water molecule), and it tackles the “macroscopic” response directly, under the form of induced polarization, which by fitting provides the linear as well as the nonlinear optical responses.^[25]^

Besides the complexities presented above, the inclusion of the environment or surrounding effects – here, the solvent – always deserves attention. Indeed, the surroundings can tune the (nonlinear) optical properties drastically. For example, the absorption spectrum of betaine dyes is employed to define a solvent polarity scale.^[26]^ In QC calculations, the most commonly employed solvation method is based on its continuous description,^[27]^ which was developed for the bulk region. Nevertheless, it has been extended to study interfaces by defining a dielectric constant that depends on the interface distance. Indeed, the water density changes as a function of the interface distance, and, therefore, the corresponding dielectric constant values change as well.^[28]^ However, this continuum description of the solvent neglects the orientational polarization induced by the explicit surrounding water molecules. Alternatively, discrete solvation methods describe the surrounding effects as electrostatic discrete local field,^[29]^ atomic point charges,^[30]^ or polarizable atomic sites,^[31]^ which are embedded in the QC calculations. Those discrete solvation methods are reliable tools to investigate the SHG responses at interfacial regions because the anisotropic surrounding effects are naturally included. However, a systematic study comparing different solvent approximations has not yet been reported for interfacial SHG responses.

In the current study, classical MD and QC methods are enacted to investigate the SHG response of the water–vacuum interface. Different approximations are used to model the effects of the surrounding on their linear and nonlinear optical properties. First, classical MD simulations are conducted to simulate a water slab, and then QC calculations are performed on sampled configurations extracted from the MD trajectories. This two‐step methodology is known as Sequential‐Quantum Mechanics/Molecular Mechanics[^32^, ^33^] or merely as multi‐scale simulations. In the first step, the water molecules are modeled by classical force fields (FFs). Two FFs are employed to assess how much the optical responses could be affected by different parameterizations. The surrounding effects in the QC calculations are then scrutinized using different approximations, going from the isolated clusters to polarizable embedding potentials. To better understand the interfacial region, the water slab was split and analyzed in layers, revealing the structural and electronic properties as a function of the interface distance.

This paper is organized as follows: We first present the results and discussions of the structural and optical properties, with the essential methodology. Next, the main conclusions are drawn. The last section presents the full description of the employed computational methods.

## Results and Discussion

### Structural Analyses

**Figure 1:**
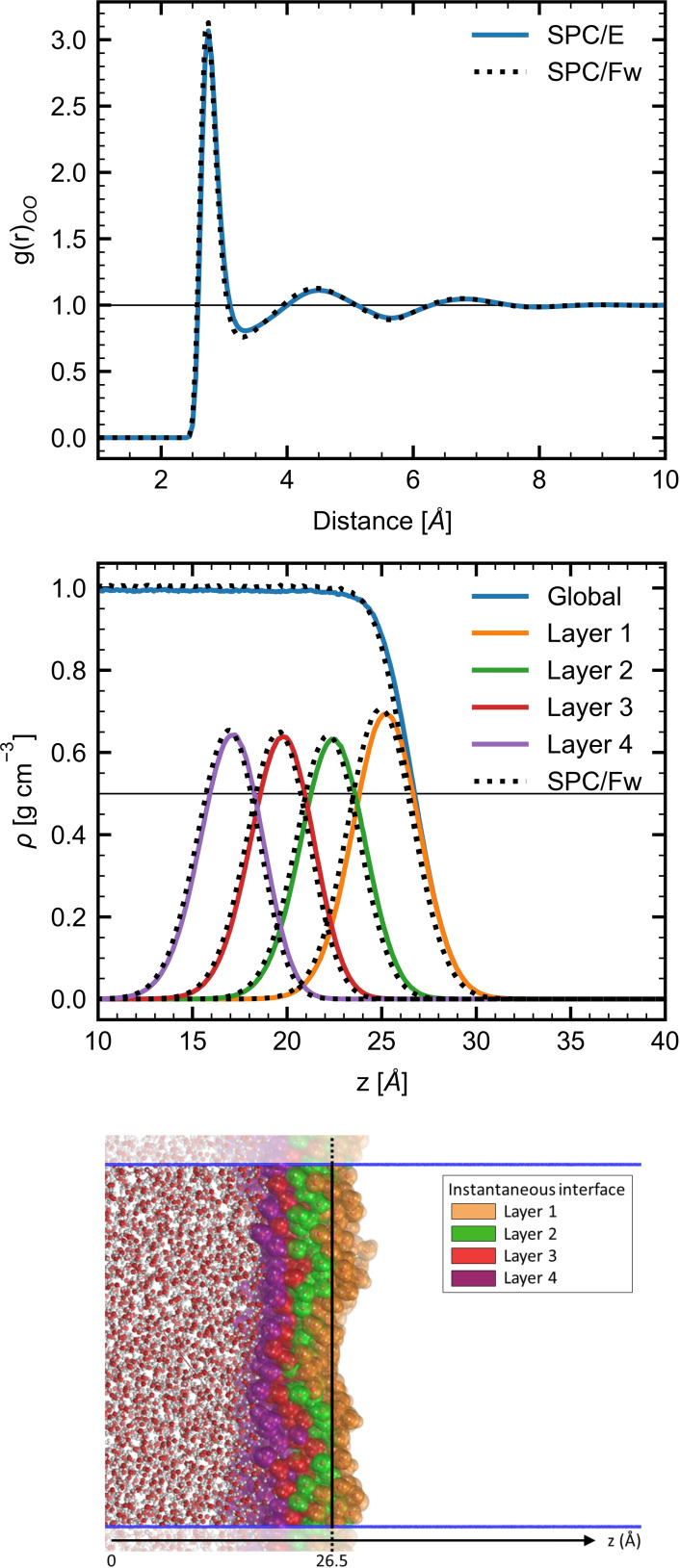
(top) Bulk oxygen–oxygen RDFs and (middle) interfacial density profiles as determined with the SPC/E (colored continuous lines) and SPC/Fw (black dashed lines) force fields. (bottom) Snapshot of the water–vacuum interface highlighting its first four molecular layers and the average interface position. Blue lines identify the MD simulation box.

Water slabs of around 50 Å thickness as well as reference bulk MD simulations were performed using the rigid SPC/E^[34]^ and flexible SPC/Fw^[35]^ force fields (details are provided in the Computational Section). In the bulk MD simulations, the Radial Distribution Function (RDF) provides the local density profile, the position of the solvation shells and their coordination numbers. Therefore, the RDFs cannot accurately describe the asymmetry at the water–vacuum interface, so that the density profile is often evaluated as a function of the surface normal direction (z‐direction). In addition to this global density profile, the corresponding density profiles associated with the four first molecular layers were evaluated using the Identification of the Truly Interfacial Molecules (ITIM)^[36]^ method. The bulk RDFs and interface density profiles are shown in Figure 1, together with a snapshot of the interface. Equivalent results were obtained using both the SPC/E and SPC/Fw force fields, which means that the general water structure is similar for both FFs. The oxygen–oxygen RDFs in the bulk, represented by g(r)_OO_, show the first maximum at 2.75 Å. The first solvation shell encompasses O−O distances up to 3.35 Å (SPC/E) and 3.30 Å (SPC/Fw) and comprises on average 4.5 (SPC/E) and 4.3 (SPC/Fw) water molecules. These similar results obtained on the first shell were also reproduced at the second (24.5 water molecules up to 5.65 Å) and third (69.0 water molecules up to 7.95 Å) shells, and they are in good agreement with previous calculations^[35]^ and measurement of the first shell position at 3.3 Å.^[37]^ The calculated bulk densities amount to 0.998±0.002 and 1.008±0.002 g cm^−3^ for SPC/E and SPC/Fw, respectively, which are in agreement with Ref. [35].

The global interfacial and layer density profiles are also similar if the SPC/E and SPC/Fw force fields are used, yet they show slightly larger densities values for the latter. The decrease of the global density and the position of the peaks on the layer density profiles occur first for MD simulation using SPC/Fw due to the larger density values, which lead to a slightly thinner water slab. The average densities obtained in the bulk‐like region (−23 Å≤z≤23 Å) of the water slab are 0.994±0.001 g cm^−3^ and 1.005±0.001 g cm^−3^ for SPC/E and SPC/Fw, respectively, which were a bit smaller than those obtained in the bulk. The position of the interface is defined by the z‐coordinate where the global density drops by half with respect to the bulk. This corresponds to a distance of 26.5 Å with respect to the center of mass of the water slab (z_*cm*_=0 Å). The maximum density of the first layer (L_1_) peaks at z=25.2 Å, with a density value of 0.69 g cm^−3^. The next three layers (i=2, 3, and 4) show similar maximum density values of 0.64 g cm^−3^, indicating a fast convergence to the bulk structure. The largest density value of L_1_ is ascribed to missing water molecules in the vacuum region, leading to stronger short‐range interactions and more compact structures. Each layer is shifted by 2.7 Å from the next one. In addition, the density profile of L_1_ presents the largest δz width or variance with a value of 1.76 Å, in comparison to 1.70, 1.66, and 1.64 Å for L_2_, L_3_, and L_4_, respectively. Again, as observed from the bulk MD simulations, the general structure was well reproduced with both rigid and flexible water FFs models.

The water molecules were indexed as belonging to L_1_‐L_4_ layers using the ITIM method, which allows distinguishing between the average intra‐ and inter‐layer HB values. The HB results are shown in Table 1. The water molecules belonging to L_1_ form more intra‐layer HB (1.9–L_1,1_) than the ones in other layers (1.3–L_i,i_ with i≠1). On the other hand, the inter‐layer HB value is slightly smaller at the L_1,2_ (0.9) interface than in deeper layers (1.0). This has been related to the large density observed in L_1_, which allows more intra‐layer HBs and creates a 2D‐HB network.^[38]^ The total HB number for the i‐th layer is defined as L_(i−1),i_+L_i,i_+L_i,(i+1)_ where L_0,1_=0 (i. e., layer 0 is the vacuum region). Using this definition and moving towards the center of the water slab, the number of HBs converges fast, showing the smallest value for L_1_ (2.8) due to the interface with the vacuum. The converged value amounts to 3.3, which is close to 3.5 as calculated for the bulk reference. As observed on the density profiles, both SPC/E and SPC/Fw provide the same average numbers of HBs.

**Table 1:** The average number of hydrogen bonds as a function of the layer (L_i_). Both SPC/E and SPC/Fw provided the same values.

i	L_i,i_	L_i,(i+1)_	Total^[a]^
1	1.9	0.9	2.8
2	1.3	1.0	3.2
3	1.3	1.0	3.3
4	1.3	1.0	3.3
Bulk	–	–	3.5

[a] The total value corresponds to the L_(i−1),i_+L_i,i_+L_i,(i+1)_ sum.

### Optical Properties

#### Selecting a Level of Approximation

**Figure 2:**
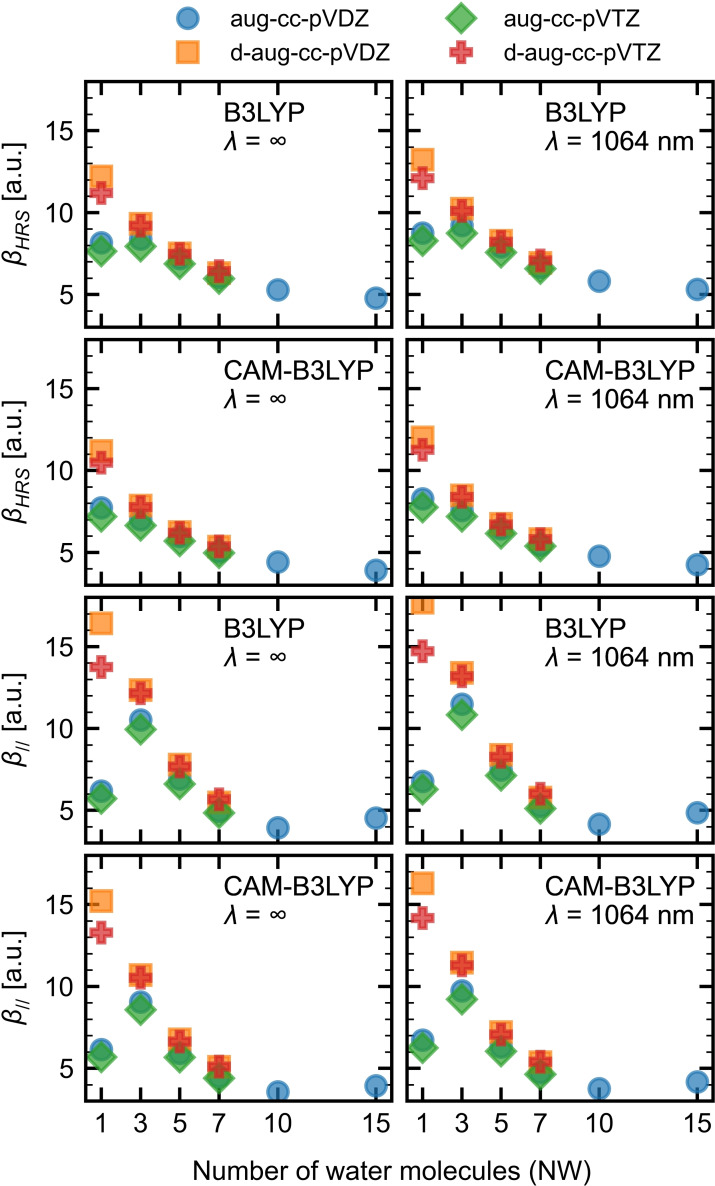
Evolution of the *β_HRS_* and *β*_*//*_. responses as a function of the number of water molecules (NW), as obtained at the TDDFT level using different XC functionals and atomic basis sets.

The Hyper‐Rayleigh Scattering (*β_HRS_*) and Electric Field Induced SHG (*β*_*//*_. responses at the center of mass of the water slab were evaluated to select a reliable level of calculation. The following parameters were considered: the basis set and the exchange‐correlation functional (XC). The impact of the number of water molecules (NW) handled at the QC level was also addressed. Both static and dynamic *α* and *β* quantities were considered, and the reported values are averages over 10 snapshots extracted from the SPC/E trajectory (Figure 2 and Table S1). The **pol‐sep** approximation (details are provided in the Computational Section) was used to include the effects of the surrounding water molecules within a 15 Å radius sphere.

The basis set effects on the *β* responses give the following ordering for their magnitude: aug‐cc‐pVTZ<aug‐cc‐pVDZ<d‐aug‐cc‐pVTZ<d‐aug‐cc‐pVDZ for both XC functionals, for static and dynamic responses, as well as for the different NW values. When there is only one water molecule in the QC core (NW=1), adding a second set of diffuse functions leads to an increase of about 50 % of *β_HRS_* and more than 150 % for *β*_*//*_. This difference drops to a maximum of 15 % for NW=5 or 7, highlighting basis set cooperative effects. Note that basis sets with a lot of diffuse functions could lead to electronic spill‐outs in the classical region because of the lack of exchange repulsion between the quantum and classical regions.^[31]^ Therefore, owing to (i) the compromise between accuracy and CPU time as well as (ii) the lower probability of electronic spill‐out, the aug‐cc‐pVDZ basis set has been chosen.

Regarding the XC functionals, B3LYP and its range‐separated analog, CAM‐B3LYP, show the same trends, although CAM‐B3LYP provides systematically smaller values. Though qualitative conclusions would be very similar, the CAM‐B3LYP XC functional has been selected because it usually provides better quantitative accuracy.^[39]^

The choice of the embedding size is as important as the choice of the QC method, and, to make it, the interfacial *β_zzz_* responses were compared for embeddings comprising water molecules within a sphere of 10, 15, and 20 Å radius centered at (0, 0, z=26.5 Å). Surrounding effects were accounted for using two different approximations, **pol‐sep** and **pol‐sep‐eef**, on a set of 100 snapshots extracted from the SPC/E trajectory. Within the **pol‐sep** model, *β_zzz_* amounts to 0.02 a.u., −0.13 a.u., and −0.12 a.u. for 10, 15, and 20 Å radius, respectively. In addition, in the same order, it attains −5.22 a.u., −5.93 a.u., and −6.21 a.u. when using the **pol‐sep‐eef** scheme. Thus, embedding considering water molecules within a 15 Å distance of the reference point gives *β_zzz_* values within 10 % of variations with respect to a larger radius (20 Å).

Both *β_HRS_* and *β*_*//*_ (per water molecule) show the same behavior as a function of the size of the water cluster: a decrease and a leveling off around 10–15. Increasing the NW in the QC core accounts for specific interactions between the water molecules, including hydrogen bonds, which are missing for NW=1. The relative orientation of the water molecules leads to partial cancellations of the dipolar and octupolar components of the *β* responses and, therefore, to a decrease of the amplitude of the *β* responses as well as of its dipolar and octupolar characters. Nevertheless, the DR and ρ values are rather constant for NW>1 because the dipolar and octupolar contributions decrease in the same way (Figure S1). Similar effects have been evidenced in the case of aggregates of π‐conjugated chromophores bearing donor/acceptor substituents.^[40]^ The larger part of this decrease is observed when going from NW=1 to NW=5, which matches to the number of molecules in the first solvation shell and the number of hydrogen bonds.

In summary, on the basis of these comparisons, the following calculations were performed at the CAM‐B3LYP/aug‐cc‐pVDZ level, while the effects of the surrounding are accounted for water molecules within a sphere of 15 Å radius. The QC core is set to five water molecules (NW=5), consistent with the average number of HBs and the number of molecules in the first solvation shell. For comparison, the experimental *β_HRS_* and *β*_*//*_ values of the water molecule amount to 9.8 a.u.^[41]^ and 12.8 a.u.^[42]^ [*β_z_* =0.46×10^−31^ esu within the X convention, which was converted to the T convention using *β*_*//*_=12/5 *β_z_*], respectively. These are larger than the calculated values. So, when considering the CAM‐B3LYP/aug‐cc‐pVDZ (λ=1064 nm) values for NW=5, this corresponds to underestimations by 34 and 51 %, respectively. It is, however, beyond the scope of this paper to assess this agreement because one should keep in mind that the experimental values are obtained in comparison to reference values as well as by employing simplified local field factors.

#### Variations of the Properties between the Bulk and the Interface as Determined with Different Embedding Models

**Figure 3:**
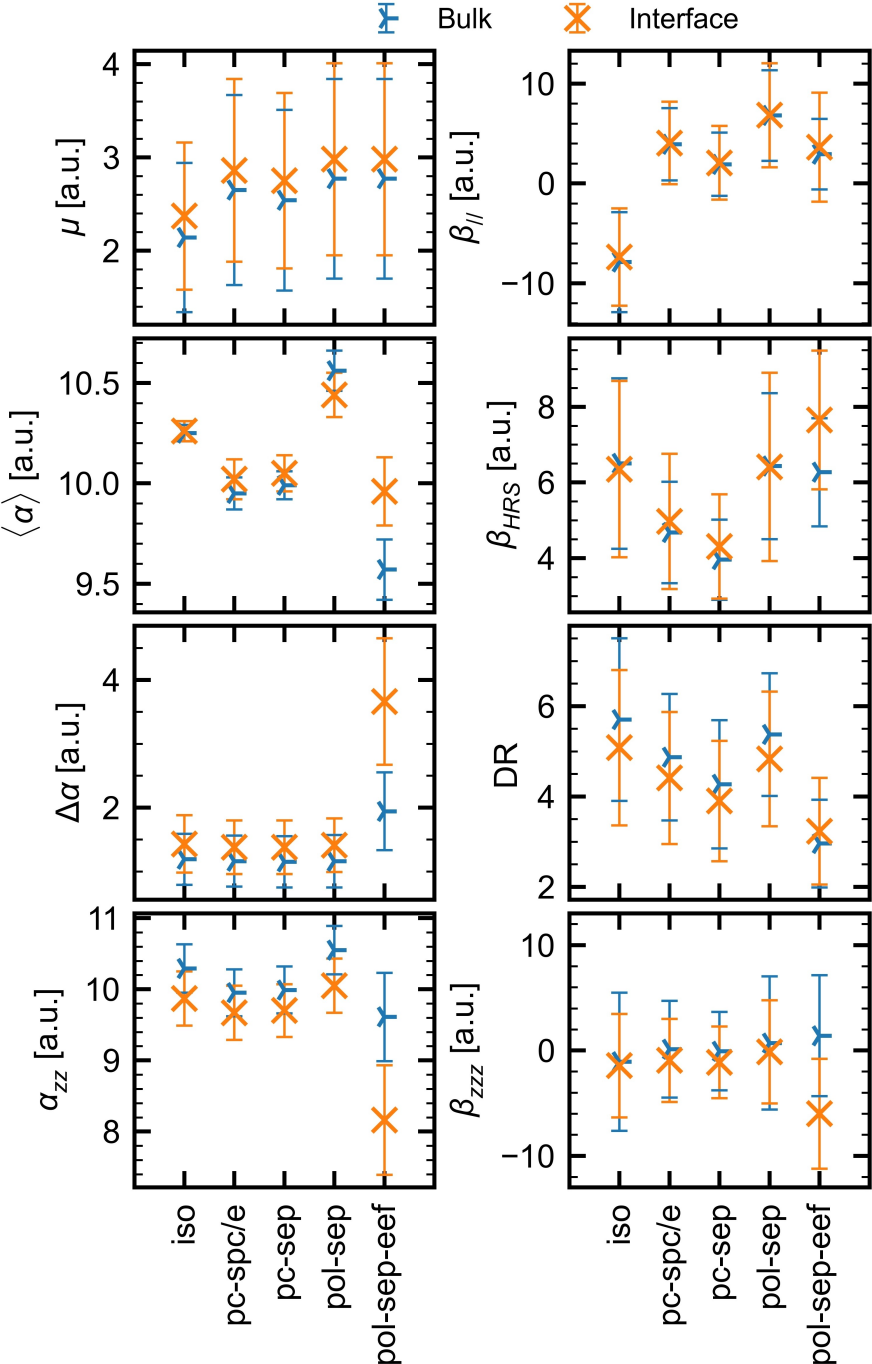
Comparison between the linear and nonlinear optical properties of the bulk and interface, as calculated at the CAM‐B3LYP/aug‐cc‐pVDZ level of approximation (λ=1064 nm for the *α* and *β* quantities) for different embedding models. The average values are represented by symbols, whereas the standard deviations are by error bars. All water molecules within a sphere of 15 Å radius were considered in the embedding approximation, except for **iso**, where no embedding was considered. The averages were performed over 100 snapshots extracted from the SPC/E trajectory.

The effects of different embedding approximations were evaluated in the bulk and interfacial regions of the water slab and the bulk reference MD simulations. The interfacial region of the water slab is defined by the NW=5 molecules closest to the (0, 0, 26.5 Å) coordinate, no matter to which molecular layer they belong, as well as the embedding comprising water molecules within a 15 Å radius sphere. The results are presented in Figure 3 (as well as in Table S2 and Figure S2), and they encompass the averages and standard deviations of 100 snapshots extracted every 300 ps. In addition, for selected yet still representative cases, Figure S3 provides the evolution of the average of the standard deviation, and of the standard error of the mean as a function of the number of snapshots. The convergence of the standard error of the mean indicates that 100 snapshots constitute a representative sample set. Figure S4 provides the analog of Figure 3 with the error bars corresponding to the standard error of the mean rather than the standard deviation, highlighting the consistency between the two pictures for drawing conclusions on the differences between the embedding models. For all quantities, the results from the reference bulk and those from the bulk‐like region of the water slab are similar (Figure S2), ensuring that the thickness of the water slab is large enough to reproduce the bulk structure at its center.

In general, the **iso**, **pc‐spc/e**, **pc‐sep**, and **pol‐sep** models slightly distinguished the bulk and interfacial regions. The two models (**pc‐spc/e** and **pc‐sep**) including the surrounding effects as atomic point charges present comparable results, tending to larger values for **pc‐spc/e** than **pc‐sep**. These results indicate that the optical properties are not strongly dependent on the atomic point charge definition of the embedding. Analogous weak dependence has been reported on the linear and nonlinear absorption spectra of *para‐*nitroaniline.[^43^, ^44^] For the **pol‐sep** model, which includes the polarization of the embedding, the values were, in general, larger than the ones obtained for the atomic point charges embeddings.

When going from the bulk to the interface, the dipole moment increases by 0.2 a.u., no matter which embedding scheme is used, which is less than 10 % of the total value. The latter amounts to about 2.8‐3.0 a.u., with the exception of the smaller (2.4 a.u.) **iso** value. In the case of polarizability, the variations are small when the water molecules move from the bulk to the interface. In the bulk, its three diagonal tensor components (*α_xx_*, *α_yy_*, and *α_zz_*) are similar, so that, ⟨α⟩ ∼*α_zz_* and Δ*α* is small. At the interface, *α_zz_* decreases while the other values increase, keeping ⟨α⟩ mostly unchanged while Δ*α* increases. This effect is however enhanced when using the **pol‐sep‐eef** approach, where *α_zz_* decreases by about 15 % while ⟨α⟩ increases by 3 % and Δ*α* is almost twice as large.

The results on the second‐order NLO responses are more contrasted and contain additional information. In the case of $\beta_{HRS}$ , the differences between the bulk and interface values are small. Except for the **pol‐sep‐eef** approach, these differences are smaller than 10 % and smaller than the standard deviations on *β_HRS_*. In that case, *β_HRS_* increases by about 20 % for the interface due to the inclusion of the effective electric field. The evolution of *β_HRS_* as a function of the model shows compensating effects: the inclusion of point charge embedding (**pc‐spc/e** and **pc‐sep**) reduces the *β_HRS_* response (with respect to **iso**) but the polarizable embedding (**pol‐sep**) restores the **iso** value. The DR values are slightly smaller at the interface and remain in the 3.9‐5.1 window, with the exception of the **pol‐sep‐eef** approach where DR increases, yet to reach a smaller value of 3.2. The changes in DR already highlight that the nature of the *β* response changes at the interface with respect to the bulk, as well as with the different embedding schemes. This is further evidenced for *β*_*//*_, which depends on selected tensor components as well as on the dipole moment orientation. First, *β*_*//*_ changes little from the bulk to the interface, except for the **pol‐sep‐eef** approach where it gets 20 % larger. Reminding that the dipole moment is almost unchanged, this effect is dominated by the variations of the *β* tensor components. On the other hand, changing the embedding model leads to drastic changes and, more importantly, considering an embedding model changes the sign of *β*_*//*_, from negative to positive. This positive *β*_*//*_ value is in agreement with the experimental value,^[42]^ as well as with calculations on water clusters by using the differential shell approach.^[45]^

Nevertheless, the *β_HRS_* and *β*_*//*_ observables were designed to describe molecules in isotropic media so that they only partially characterize the *β* changes from the bulk to the interface. So, the *β_zzz_* component, which dominates the tensor, is further indicative. Using the **pol‐sep‐eef** approach, *β_zzz_* in the bulk can be considered negligible because the average value is much smaller than its standard deviation, whereas it is clearly non‐zero (and negative) at the interface, highlighting the preferential ordering of the water molecules at the interface. On the other hand, the other embedding models, as well as the isolated one, are not sufficient to evidence an interfacial effect because the average values and their bulk–interface variations are small with respect to the standard deviations.

#### Evolution of the Properties from the Interface to the Bulk

**Figure 4:**
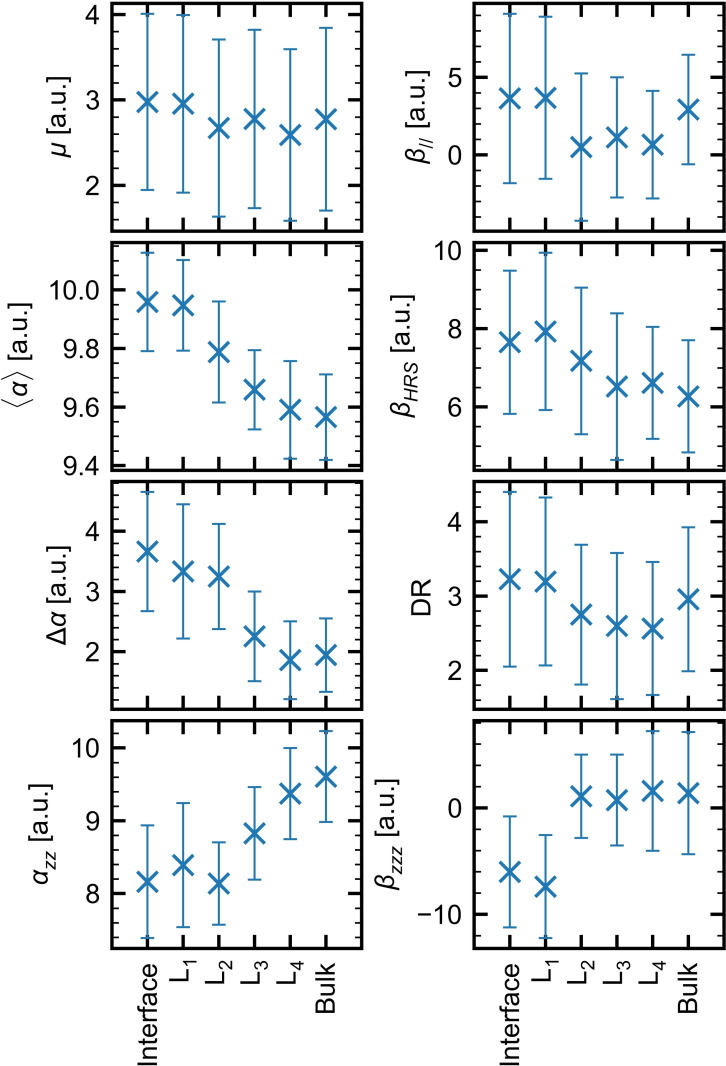
Comparison between the linear and nonlinear optical properties for different molecular layers, as calculated at the CAM‐B3LYP/aug‐cc‐pVDZ level of approximation (λ=1064 nm for the *α* and *β* quantities). The average values are represented by symbols, whereas the standard deviations are by error bars. All water molecules within a sphere of 15 Å radius were considered in the **pol‐sep‐eef** approximation. The averages were performed over 100 snapshots extracted from the SPC/E trajectory.

For the interface region, the QC core is defined based on the five first water molecules close to the (0, 0, z=26.5 Å) position, no matter to which layer they belong. In contrast, selecting the NW molecules belonging to a given molecular layer requires extra constraints than those imposed in the so‐called interface region, where only distance criteria were imposed. For example, almost 20 % of the water molecules in the interface region do not belong to the first layer, and this can be observed on the representative 15 ns snapshot given in Figure S5. Then, by construction, the L_i_ water clusters encompass the five water molecules belonging to the i‐th layer and which are the closest to the (0, 0, z_i_) coordinate. The z_i_ values are 25.2, 22.4, 19.8, and 17.3 Å, which correspond to the density profiles maxima obtained for L_1_, L_2_, L_3_, and L_4_, respectively (Figure 1). As shown in Figure S5, imposing the NW molecules to belong to a given layer increases the two‐dimensional (2D) character of water clusters, even though the clusters are not flat. As the **pol‐sep‐eef** model was the unique one to distinguish the *β_zzz_* values between the interface and the bulk, it is used to monitor the evolution of the properties from the interface to the bulk. The results are presented in Figure 4 and Table S3.

The dipole moment values are similar to each other. They range from 3.0 to 2.6 a.u. when going from the interface to L_4_. Moreover, from L_2_ to the bulk, μ remains about 2.7 a.u., indicating the bulk value is rapidly reached. Contrary to the dipole moment, the polarizability shows a clear evolution as a function of the molecular layer, yet with clear leveling off towards the bulk value when reaching L_4_. So, ⟨α⟩ evolves smoothly from 10.0 a.u. at the very interfacial regions (interface and L_1_) to 9.6 a.u. in L_4_ and in the bulk. A smooth behavior is also observed for the $\alpha_{zz}$ tensor component, though it increases when going towards the bulk. Though not shown here, *α_xx_* and *α_yy_* tensor components adopt the opposite trend, the same as ⟨α⟩ , in such a way that the anisotropy decreases by a factor of two from the interface to the bulk. As a matter of comparison, Osted et al.^[46]^ have fitted previously measured[^47^, ^48^] refractive indices of liquid water to obtain a first‐order susceptibility value of $\chi^{\left(1\right)}$ =0.060 at λ=1064 nm. This leads to ⟨α⟩ =10.12 a.u., which is slightly larger than our bulk value.

The variations on *β* are, to a certain extent, less monotonic and depend on the type of second‐order response. On the one hand, *β_HRS_* decreases by about 20 % from the interface to the bulk. Yet, the L_1_ value is larger than the interface response but their standard deviations being of the order of 25 %, it is safer to conclude that there is simply a decrease from the interface to the bulk. This *β_HRS_* is characterized by rather stable DR values over the successive layers. In the case of *β*_*//*_, the results tend to show that it decreases from L_1_ to the inner layers and the bulk, but this effect is weak, considering the standard deviations. This analysis is substantiated by the *β_zzz_* values, of which the amplitude (*β_zzz_* is negative for L_1_ and the interface) drops from the interface to the bulk, where it is negligible. In summary, no interfacial effects can be observed for the second‐order NLO responses beyond the first molecular layer, while the effects on the linear optical responses are observed up to the fourth molecular layer.

In addition to the results obtained using the MD trajectories with the rigid SPC/E water FF, extra QC calculations using the flexible SPC/Fw water FF trajectories were carried out to assess the impact of the water structure on the linear and nonlinear optical properties. In most cases, the (N)LO results obtained from the SPC/Fw trajectories are similar to the ones obtained from the SPC/E trajectories. Moreover, the relationships between these two FFs are described by straight lines and similar trends from bulk to the interface. In addition, the reference bulk and the bulk‐like region of the water slab provide equivalent results (Figure S6).

## Conclusion and Outlook

A systematic study of the polarizability (*α*) and first hyperpolarizability (*β*) responses at the water‐vacuum interface has been carried out by adopting a two‐step approach known as the Sequential Quantum Mechanics(QM)/Molecular Mechanics(MM) scheme, where classical molecular dynamics simulations are performed to provide snapshots, which are then employed to evaluate the linear and nonlinear optical responses at a quantum chemistry (QC) level. In the latter step, besides calculations on isolated clusters, different embedding schemes are adopted, ranging from point charges to polarizable point charges, with and without local field effects in order to account for the solvent/surrounding effects. The QC calculations of the *α* and *β* responses have been performed at the time‐dependent DFT level, with the CAM‐B3LYP XC functional and the aug‐cc‐pVDZ basis set. These have been carried out on small clusters containing five water molecules as representative of the QC region. In particular, the study highlights how the *α* and *β* responses evolve when going from the interface to the bulk.

So, from the interface to the bulk, the average polarizability decreases by a few percent (4 %) while the polarizability anisotropy decreases by a factor of two. This behavior results from the fact that the diagonal *α* tensor components get more similar when going towards the bulk. In the case of the second‐order NLO responses, *β_HRS_* is about 20 % larger at the interface, with small variations of its depolarization ratio. On the other hand, the effects are stronger on the component of the *β* tensor normal to the interface (*β_zzz_*) that vanishes when going from the interface or the first layer towards the bulk. Though its variations are partially masked by the large standard deviations, the *β_II_* values follow the same trend as *β_zzz_*. Globally, the interfacial effects on the second‐order NLO responses are localized at the first molecular layer, while the effects on the linear responses are observed up to the fourth molecular layer. Calculations have also shown that the effects of the surrounding on the *α* and *β* responses of water at the interface are non‐negligible and that simplified models, which do not account for polarizable point charges and effective fields, are lacking a part of these effects. After this investigation of water molecules in water, further works will be devoted to the linear and nonlinear responses of small molecules like methanol and ethanol in water as well as to the role of anions and cations.

## Computational Section

### Molecular Dynamics Simulations

Molecular dynamics (MD) simulations were performed to obtain snapshots of the water structure in the bulk and at the interface, aiming for describing the variations of density and orientation as a function of the distance to the interface. All MD simulations were performed with the GROMACS[^49^, ^50^] software. The water molecules were modeled by the rigid SPC/E^[34]^ force field (r_OH_=1.0 Å, θ_HOH_=109.47°, $\sigma_{O}$ =3.165 Å, $\epsilon_{O}$ =0.155 kcal mol^−1^, q_O_=−0.8476 e, q_H_=0.4238 e), and they were kept rigid by using the SETTLE^[51]^ algorithm. An initial cubic box of 53 Å edge size was filled out randomly with 5000 water molecules. To reduce the forces on this non‐equilibrated initial configuration, an energy minimization procedure was performed using the steepest descent algorithm with a 350 kJ mol^−1^ nm^−1^ tolerance and 3D periodic conditions. Then, an NVT thermalization of 2.5 ns was performed to allow the system to reach a thermalized configuration at 300 K. This NVT thermalization was carried out by generating initial velocities based on the Maxwell distribution at 300 K; the MD simulations were performed using the leap‐frog algorithm^[52]^ every 1 fs timestep; the temperature was controlled by the velocity rescaling thermostat^[53]^ every 100 fs; the short‐range nonbonding interactions were computed within a sphere of 14 Å radius; and, the long‐range electrostatic corrections were accounted for by the smooth particle‐mesh Ewald method.^[54]^ At the end of the thermalization, the final configuration was named as the bulk thermalized box configuration, and it was used as the initial condition for the following MD simulations. On the one hand, an NPT reference bulk production of 30 ns was performed after an extra 2.5 ns long NPT thermalization, which was carried out in the same manner as in the previous NVT thermalization but additionally coupling the Berendsen^[55]^ barostat every 1 ps. On the other hand, the z‐edge of the bulk thermalized box configuration was increased to 200 Å, creating a water slab with two water‐vacuum interfaces. This ≈150 Å of vacuum is large enough to avoid any interactions between the water slab and its corresponding ±z‐images of the 3D periodic system. In addition, these two vacuum layers allow the water slab to freely expand or compress due to the asymmetrical molecular interactions. As a result, an infinite water slab of ≈50 Å thickness was simulated. This new z‐elongated box was then thermalized using the NVT thermalization approach previously described, except for the initial velocities, which correspond to those of the bulk thermalized box configuration. Finally, the analyzed snapshots were extracted from an NVT production step of 30 ns.

To investigate possible force field dependence on (nonlinear) optical responses, additional MD simulations were performed using the flexible SPC/Fw^[35]^ force field (r_OH_=1.012 Å, *k*_r_=1059.162 kcal mol^−1^ Å^−2^, θ_HOH_=109.47°, k_θ_=75.90 kcal mol^−1^ rad^−2^, $\sigma_{O}$ =3.165 Å, $\epsilon_{O}$ =0.155 kcal mol^−1^, q_O_=−0.82 e, q_H_=0.41 e). Except for the integration time, which was 0.5 fs for the flexible model (instead of 1 fs for the rigid one), all the other parameters of the SPC/E MD simulations were employed for the SPC/Fw ones. Thus, the SPC/E configuration was the initial condition to a 2.5 ns long thermalization using the SPC/Fw FF for both bulk (NPT) reference and water‐vacuum interface (NVT). Then, 30 ns of production trajectories were evaluated in the bulk (NPT) reference and water‐vacuum interface (NVT).

### Structural Analyses

The structural properties of the water in the bulk and at the interface were described in terms of two principal parameters: the density profiles and the average number of hydrogen bonds. In the bulk, the Radial Distribution Function (RDF) defines the local density profile in reference to a given atom. Moreover, it is possible to describe the position of the solvation shells and their coordination numbers. The RDFs analyses were performed using the GROMACS analysis tools. Close to the water‐vacuum interface, the density profile was evaluated as a function of the position along the z‐direction, providing therefore an estimate for the average interface position. On the other hand, however, it was not possible to evaluate neither the roughness of the surface nor to assign the water molecules to different layers.

To further understand the interfacial structure, the Identification of the Truly Interfacial Molecules (ITIM)^[36]^ scheme was enacted using the Pytim^[56]^ python package and the MDAnalysis[^57^, ^58^] library. The ITIM method consists of moving probe spheres, in the z direction, with a radius of 1.5 Å from the vacuum region towards the z‐center of the water slab. The probe spheres stop always they touch a water oxygen atom (with $\sigma_{O}$ /2 radius), defining an instantaneous surface. Thus, the water molecules in contact with the probe spheres were assigned as belonging to the first layer (L_1_). Then, the L_1_ molecules were removed, and the probe spheres moved again towards the z‐center of the water slab, defining the second layer (L_2_). This procedure was repeated 4 times, indexing the water molecules in 4 layers. The full description of the method is provided in Refs. [36, 56].

Another helpful analysis to understand the structure of water is to assess the number of hydrogen bonds (HBs). The oxygen‐oxygen donor‐acceptor distance d_DA_≤3.5 Å and donor‐hydrogen‐acceptor angle θ_DHA_≥140.0° cutoffs were defined as the HB geometrical criteria. Indexing the water molecules in layers allows evaluating the properties as a function of the layer, that is, the intra‐layer HBs. The average number of HBs for molecules in the same layer and molecules belonging to the next layers were evaluated from 60000 snapshots. Due to the dynamics of the water molecules along the simulation time, the water molecules can switch from one layer to another, generating difficulties to calculate the HB average values. To overcome these, an arbitrary water molecule belonging to the target layer was selected as a reference for each snapshot. All HB analyses were performed using the MDAnalysis library.

### Optical Properties

The optical properties of molecules are defined by the expansion of the induced dipole moment ($\Delta \vec{\mu }$ ) as a function of perturbative electric fields ($\vec{E}$ ) oscillating at ω angular frequencies. The polarizability (*α*) and first hyperpolarizability (*β*) are tensors of rank 2 and 3, respectively. In the T convention, they contribute to $\Delta \vec{\mu }$ by the following expression [Eq. 1]:

(1) $$\Delta \mu_{i}\left(-\omega_{\sigma }\right)=\;\sum_{j}^{x,y,z}\alpha_{ij}\left(-\omega_{\sigma };\omega_{1}\right)E_{j}\left(\omega_{1}\right)+\frac{1}{2!}\;\sum_{j,k}^{x,y,z}\beta_{ijk}\left(-\omega_{\sigma };\omega_{1},\omega_{2}\right)E_{j}\left(\omega_{1}\right)E_{k}\left(\omega_{2}\right)+\;\cdots$$

where $\omega_{\sigma }=\sum_{n}\omega_{n}$ and the lower‐case indices define the molecular axes coordinates (x,y,z ).^[59]^ For *α*, the tensor elements can be combined to provide the mean ⟨α⟩ and the anisotropy Δ*α* invariants [Eqs. (2) and 3]:

(2) $$\left\langle \alpha \right\rangle =\frac{1}{3}\;\sum_{i}^{x,\;y,\;z}\alpha_{ii}$$

(3) $$\Delta \alpha =\sqrt{\frac{1}{2}\;\sum_{i,j}^{x,y,z}\left(3\alpha_{ij}^{2}-\;\alpha_{ii}\alpha_{jj}\right)}$$

For describing *β* of molecules in isotropic media, the Hyper‐Rayleigh Scattering (HRS) technique^[13]^ is an efficient method. From the SHG scattered light intensities, it provides $\beta_{HRS}\left(-2\omega ;\omega ,\omega \right)$ [Eq. (4)], as well as its components, the vertical‐vertical ($\left\langle \beta_{ZZZ}^{2}\right\rangle$ , Eq. (5)) and horizontal‐vertical ($\left\langle \beta_{ZXX}^{2}\right\rangle$ , Eq. (6)) invariants.

(4) $$\beta_{HRS}\left(-2\omega ;\omega ,\omega \right)=\beta_{HRS}=\sqrt{\left\langle \beta_{ZZZ}^{2}\right\rangle +\left\langle \beta_{ZXX}^{2}\right\rangle }$$

(5) $$\left\langle \beta_{ZZZ}^{2}\right\rangle =\frac{1}{105}\sum_{i,j,k}^{x,y,z}\left[2\beta_{ijk}^{2}+\beta_{ijj}\beta_{ikk}+4\left(\beta_{iij}\beta_{jkk}+\beta_{iij}\beta_{kkj}+\beta_{ijk}\beta_{jik}\right)\right]$$

(6) $$\left\langle \beta_{ZXX}^{2}\right\rangle =\frac{1}{105}\sum_{i,j,k}^{x,y,z}\left[6\beta_{ijk}^{2}+3\beta_{ijj}\beta_{ikk}-2\left(\beta_{iij}\beta_{jkk}+\beta_{iij}\beta_{kkj}+\beta_{ijk}\beta_{jik}\right)\right]$$

Additionally, the Depolarization Ratio (DR) provides information on the shape of the NLO scattered moiety, being perfectly octupolar for DR=1.5 and perfectly dipolar for DR=9.0. DR values within these extremes correspond to combinations of octupolar and dipolar characteristics. The DR value is obtained as the ratio [Eq. 7]:

(7) $$DR=\frac{\left\langle \beta_{ZZZ}^{2}\right\rangle }{\left\langle \beta_{ZXX}^{2}\right\rangle }\;$$

*β_HRS_* can also be expressed in terms of its irreducible spherical representations.^[14]^ In the static case, it reads [Eq. 8]:

(8) $$\beta_{HRS}=\sqrt{\frac{10}{45}\beta_{J=1}^{2}+\frac{10}{105}\beta_{J=3}^{2}}$$

where *β*_*J=1*_ and *β*_*J=3*_ are the dipolar and octupolar contributions, of which the ratio determines the nonlinear anisotropy parameter [Eq. 9].

(9) $$\rho =\frac{\beta_{J=3}}{\beta_{J=1}}$$

Electric Field‐Induced Second Harmonic Generation (EFISHG) is another experimental technique often used to obtain the *β* response in a liquid phase. The EFISHG probes the vector representation of *β* projected on the dipole moment direction [Eqs. (10) and 11].

(10) $$\beta_{//}\left(-2\omega ;\omega ,\omega \right)=\beta_{//}=\frac{3}{5\mu }\;\vec{\mu }\cdot \vec{\beta }$$

(11) $$\beta_{i}=\frac{1}{3}\sum_{j}^{x,y,z}\left(\beta_{ijj}+\beta_{jij}+\beta_{jji}\right)$$

All results are given in a.u. 1 a.u. of *α*=1.648×10^−41^ C^2^ m^2^ J^−1^=0.14818 Å^3^; 1 a.u. of *β*=3.6212×10^−42^ m^4^ V^−1^=3.2064×10^−53^ C^3^ m^3^ J^−2^=8.639×10^−33^ esu.

### Quantum Chemistry Calculations

All Quantum Chemistry (QC) calculations were performed using the Time‐Dependent Density Functional Theory (TDDFT) at the linear and quadratic response approaches, as implemented in the Dalton^[60]^ software. The surrounding effects were modeled using the Polarizable Embedding Library.^[61]^ To describe the surrounding effects on the (nonlinear) optical responses, different embedding schemes were employed to model the water surrounding: i) **pc‐spc/e** – atomic point charges based on the SPC/E force field, ii) **pc‐sep** – atomic point charges (q_O_=−0.67444 e, q_H_=0.33722 e) based on the Solvent Embedding Potential^[62]^ (SEP), iii) **pol‐sep** – atomic point charges (q_O_=−0. 67444 e, q_H_=0.33722 e) and atom localized polarizabilities (*α*_O_=5.73935 a.u., *α*_H_=2.30839 a.u.) based on SEP, iv) **pol‐sep‐eef** – atomic point charges and atom‐localized polarizabilities based on SEP and corrected with the Effective External Field,^[63]^ and v) **iso** – no water molecules were included in the embedding so that the properties were evaluated on isolated clusters (QC core) containing NW water molecules. The embeddings considered all molecules within a radius centered at the (0, 0, z) coordinate, where the origin corresponds to the center of mass of the water slab.

To define the QC level, an initial evaluation of the basis sets (aug‐cc‐pVDZ, aug‐cc‐pVTZ, and their respective double augmented analogs), exchange‐correlation DFT functionals (B3LYP, CAM‐B3LYP), and the number of water molecules in the QC core (NW from 1 to 15) was performed on 10 snapshots extracted every 3 ns from the SPC/E trajectory. A 15 Å **pol‐sep** embedding was always included in these initial analyses. The convergence of the embedding size was evaluated at the interface for a total of 100 snapshots extracted every 300 ps from the MD simulations, where the embedding effects were modeled by **pol‐sep** and **pol‐sep‐eef** for 10, 15, and 20 Å. Based on these initial analyses (see the “Results and Discussion – Selecting a Level of Approximation” sub‐section), all the remaining (nonlinear) optical properties were obtained at the CAM‐B3LYP/aug‐cc‐pVDZ level considering NW=5 water molecules in the QC core and a 15 Å radius spherical embedding for a set of 100 extracted snapshots. One water cluster comprising 5 molecules, of which the position is given by (0, 0, z) with different values of z (see the “Evolution of the Properties from the Interface to the Bulk” sub‐section) as a function of the layer, was extracted from each snapshot. All polarizability and first hyperpolarizability values are reported per water molecule, that is, as the total value divided by the number of molecules in the QC core.

## Conflict of interest

The authors declare no conflict of interest

1

## Supporting information

As a service to our authors and readers, this journal provides supporting information supplied by the authors. Such materials are peer reviewed and may be re‐organized for online delivery, but are not copy‐edited or typeset. Technical support issues arising from supporting information (other than missing files) should be addressed to the authors.

Supporting Information

## Data Availability

The data that support the findings of this study are available from the corresponding author upon reasonable request.
