# Supplementary material for: Investigation of the Second Harmonic Generation at the Water–Vacuum Interface by Using Multi‐Scale Modeling Methods
Source: ChemistryOpen. 2022 Aug 11;12(1):e202200045. doi: 10.1002/open.202200045 (PMC9806952; doi:10.1002/open.202200045)
Supplement: Supplementary file 1 — Supporting Information [file OPEN-12-e202200045-s001.pdf]

ChemistryOpen

Supporting Information

Investigation of the Second Harmonic Generation at the Water–Vacuum Interface by Using Multi-Scale Modeling Methods

Tárcius N. Ramos* and Benoît Champagne

Table S1. Averages and standard deviations (in parentheses) of the β_{HRS} and $\beta_{//}$ responses (in a.u.) as a function of the number *(NW)* of water molecules, as obtained at the TDDFT level using different XC functionals and atomic basis sets. All water molecules within a sphere of 15 Å radius were considered in the **pol-sep** embedding approximation. The averages were performed over 10 snapshots extracted from the SPC/E trajectory.

β_{HRS} – B3LYP

	$\lambda = \infty$				$\lambda = 1064$ nm			
NW	aug-cc-pVDZ	d-aug-cc-pVDZ	aug-cc-pVTZ	d-aug-cc-pVTZ	aug-cc-pVDZ	d-aug-cc-pVDZ	aug-cc-pVTZ	d-aug-cc-pVTZ
1	8.16 (1.70)	12.21 (4.02)	7.64 (2.12)	11.19 (3.98)	8.75 (1.93)	13.22 (4.51)	8.28 (2.42)	12.10 (4.47)
3	8.38 (3.73)	9.32 (3.72)	7.94 (3.63)	9.20 (3.68)	9.21 (4.31)	10.23 (4.30)	8.74 (4.19)	10.11 (4.26)
5	7.20 (1.99)	7.51 (2.20)	6.88 (1.91)	7.47 (2.05)	7.91 (2.28)	8.27 (2.51)	7.57 (2.20)	8.22 (2.34)
7	6.05 (1.66)	6.30 (1.67)	5.96 (1.59)	6.42 (1.70)	6.65 (1.86)	6.93 (1.90)	6.57 (1.80)	7.08 (1.93)
10	5.29 (1.55)	- (-)	- (-)	- (-)	5.82 (1.77)	- (-)	- (-)	- (-)
15	4.78 (1.53)	- (-)	- (-)	- (-)	5.32 (1.73)	- (-)	- (-)	- (-)

β_{HRS} – CAM-B3LYP

	$\lambda = \infty$				$\lambda = 1064$ nm			
NW	aug-cc-pVDZ	d-aug-cc-pVDZ	aug-cc-pVTZ	d-aug-cc-pVTZ	aug-cc-pVDZ	d-aug-cc-pVDZ	aug-cc-pVTZ	d-aug-cc-pVTZ
1	7.72 (1.58)	11.19 (3.49)	7.19 (1.92)	10.48 (3.54)	8.28 (1.80)	12.02 (3.86)	7.76 (2.17)	11.27 (3.94)
3	7.00 (2.66)	7.86 (2.70)	6.64 (2.64)	7.77 (2.67)	7.57 (2.99)	8.48 (3.03)	7.18 (2.94)	8.38 (2.99)
5	5.97 (1.52)	6.24 (1.70)	5.69 (1.47)	6.20 (1.60)	6.45 (1.69)	6.75 (1.88)	6.16 (1.64)	6.71 (1.78)
7	5.04 (1.31)	5.36 (1.31)	4.97 (1.22)	5.36 (1.30)	5.44 (1.42)	5.81 (1.43)	5.38 (1.34)	5.81 (1.43)
10	4.42 (1.27)	- (-)	- (-)	- (-)	4.77 (1.41)	- (-)	- (-)	- (-)
15	3.91 (1.26)	- (-)	- (-)	- (-)	4.26 (1.40)	- (-)	- (-)	- (-)

$\beta_{//}$ – B3LYP

	$\lambda = \infty$				$\lambda = 1064$ nm			
NW	aug-cc-pVDZ	d-aug-cc-pVDZ	aug-cc-pVTZ	d-aug-cc-pVTZ	aug-cc-pVDZ	d-aug-cc-pVDZ	aug-cc-pVTZ	d-aug-cc-pVTZ
1	6.18 (4.26)	16.41 (6.41)	5.71 (4.26)	13.75 (6.47)	6.78 (4.66)	17.68 (7.09)	6.29 (4.72)	14.71 (7.15)
3	10.52 (5.23)	12.35 (4.95)	9.94 (5.16)	12.15 (4.91)	11.48 (6.00)	13.40 (5.69)	10.83 (5.91)	13.19 (5.64)
5	6.91 (5.95)	7.79 (6.38)	6.61 (5.66)	7.68 (6.37)	7.47 (6.62)	8.39 (7.09)	7.13 (6.30)	8.27 (7.08)
7	4.94 (5.92)	5.48 (6.02)	4.84 (5.76)	5.67 (6.24)	5.24 (6.56)	5.78 (6.69)	5.11 (6.40)	6.02 (6.94)
10	3.95 (5.22)	- (-)	- (-)	- (-)	4.16 (5.82)	- (-)	- (-)	- (-)
15	4.53 (4.64)	- (-)	- (-)	- (-)	4.85 (5.26)	- (-)	- (-)	- (-)

$\beta_{//}$ – CAM-B3LYP

	$\lambda = \infty$				$\lambda = 1064$ nm			
NW	aug-cc-pVDZ	d-aug-cc-pVDZ	aug-cc-pVTZ	d-aug-cc-pVTZ	aug-cc-pVDZ	d-aug-cc-pVDZ	aug-cc-pVTZ	d-aug-cc-pVTZ
1	6.14 (3.98)	15.22 (5.64)	5.69 (3.93)	13.29 (5.84)	6.72 (4.34)	16.29 (6.20)	6.24 (4.34)	14.17 (6.40)
3	9.04 (3.81)	10.69 (3.73)	8.58 (3.83)	10.53 (3.65)	9.72 (4.26)	11.45 (4.17)	9.21 (4.27)	11.29 (4.09)
5	5.92 (4.84)	6.77 (5.23)	5.67 (4.62)	6.63 (5.22)	6.31 (5.28)	7.21 (5.70)	6.04 (5.04)	7.05 (5.69)
7	4.48 (4.76)	5.09 (4.97)	4.39 (4.66)	5.10 (5.05)	4.74 (5.17)	5.37 (5.41)	4.62 (5.07)	5.40 (5.51)
10	3.56 (4.30)	- (-)	- (-)	- (-)	3.76 (4.69)	- (-)	- (-)	- (-)
15	3.94 (3.72)	- (-)	- (-)	- (-)	4.18 (4.12)	- (-)	- (-)	- (-)

Table S2. Averages and standard deviations (in parentheses) of μ, $\langle\alpha\rangle$, $\Delta\alpha$, α_{zz}, $\beta_{//}$, β_{HRS}, β_{zzz} (in a.u.) and DR for the different phases (bulk reference, bulk, and interface) as a function of the embedding method. These properties were calculated at the CAM-B3LYP/aug-cc-pVDZ level of approximation ($\lambda = 1064$ nm for the α and β quantities). All water molecules within a sphere of 15 Å radius were considered in the embedding approximation, except for **iso**, where no embedding was considered. The averages were performed over 100 snapshots extracted from the SPC/E trajectory.

	μ			$\langle\alpha\rangle$		
	Bulk-ref	Bulk	Interface	Bulk-ref	Bulk	Interface
**iso**	2.17 (0.74)	2.14 (0.80)	2.37 (0.79)	10.25 (0.05)	10.25 (0.04)	10.26 (0.05)
**pc-spce**	2.69 (0.93)	2.65 (1.02)	2.86 (0.98)	9.95 (0.08)	9.95 (0.08)	10.02 (0.10)
**pc-sep**	2.58 (0.89)	2.54 (0.97)	2.75 (0.94)	9.99 (0.07)	9.99 (0.07)	10.05 (0.09)
**pol-sep**	2.82 (0.97)	2.77 (1.07)	2.98 (1.03)	10.56 (0.09)	10.56 (0.10)	10.44 (0.11)
**pol-sep-eef**	2.82 (0.97)	2.77 (1.07)	2.98 (1.03)	9.64 (0.33)	9.57 (0.15)	9.96 (0.17)

	$\Delta\alpha$			α_{zz}		
	Bulk-ref	Bulk	Interface	Bulk-ref	Bulk	Interface
**iso**	1.27 (0.46)	1.19 (0.40)	1.43 (0.45)	10.23 (0.41)	10.29 (0.34)	9.87 (0.38)
**pc-spce**	1.24 (0.41)	1.16 (0.40)	1.38 (0.42)	9.94 (0.42)	9.95 (0.33)	9.67 (0.38)
**pc-sep**	1.23 (0.42)	1.15 (0.40)	1.38 (0.42)	9.98 (0.41)	9.99 (0.33)	9.70 (0.37)
**pol-sep**	1.23 (0.41)	1.16 (0.41)	1.41 (0.42)	10.56 (0.43)	10.55 (0.34)	10.05 (0.38)
**pol-sep-eef**	1.84 (0.60)	1.94 (0.61)	3.66 (0.99)	9.60 (0.63)	9.61 (0.62)	8.16 (0.77)

	$\beta_{//}$			β_{HRS}		
	Bulk-ref	Bulk	Interface	Bulk-ref	Bulk	Interface
**iso**	-8.34 (5.08)	-7.89 (5.01)	-7.38 (4.89)	6.71 (2.45)	6.50 (2.25)	6.35 (2.33)
**pc-spce**	3.38 (3.67)	3.91 (3.62)	4.05 (4.14)	4.54 (1.56)	4.68 (1.34)	4.97 (1.79)
**pc-sep**	1.39 (3.35)	1.92 (3.16)	2.06 (3.70)	3.96 (1.28)	3.96 (1.06)	4.31 (1.38)
**pol-sep**	6.19 (4.44)	6.80 (4.53)	6.84 (5.21)	6.16 (2.02)	6.43 (1.93)	6.41 (2.49)
**pol-sep-eef**	2.16 (3.42)	2.92 (3.53)	3.64 (5.46)	6.17 (1.33)	6.27 (1.43)	7.65 (1.83)

	DR			β_{zzz}		
	Bulk-ref	Bulk	Interface	Bulk-ref	Bulk	Interface
**iso**	5.65 (1.94)	5.70 (1.80)	5.08 (1.72)	0.64 (6.49)	-1.07 (6.56)	-1.44 (4.91)
**pc-spce**	4.62 (1.47)	4.87 (1.40)	4.41 (1.46)	0.31 (4.50)	0.12 (4.59)	-0.95 (3.94)
**pc-sep**	4.12 (1.49)	4.27 (1.42)	3.90 (1.33)	0.35 (4.00)	-0.07 (3.73)	-1.13 (3.41)
**pol-sep**	5.06 (1.46)	5.37 (1.36)	4.83 (1.49)	0.31 (6.13)	0.70 (6.32)	-0.13 (4.90)
**pol-sep-eef**	2.90 (1.04)	2.96 (0.97)	3.23 (1.18)	0.94 (5.52)	1.39 (5.74)	-6.01 (5.22)

Table S3. Averages and standard deviations (in parentheses) of μ, $\langle\alpha\rangle$, $\Delta\alpha$, α_{zz}, $\beta_{//}$, β_{HRS}, β_{zzz} (in a.u.) and DR as a function of the position in the water slab (interface, L_1-L_4, and bulk-like). These properties were calculated at the CAM-B3LYP/aug-cc-pVDZ level of approximation ($\lambda = 1064$ nm for the α and β quantities). All water molecules within a sphere of 15 Å radius were considered in the **pol-sep-eef** approximation. The averages were performed over 100 snapshots extracted from the SPC/E trajectory.

	μ	$\langle\alpha\rangle$	$\Delta\alpha$	α_{zz}	$\beta_{//}$	β_{HRS}	DR	β_{zzz}
**Interface**	2.98 (1.03)	9.96 (0.17)	3.66 (0.99)	8.16 (0.77)	3.64 (5.46)	7.65 (1.83)	3.23 (1.18)	-6.01 (5.22)
**L_1**	2.95 (1.04)	9.95 (0.15)	3.33 (1.11)	8.39 (0.85)	3.67 (5.23)	7.93 (2.01)	3.20 (1.13)	-7.40 (4.84)
**L_2**	2.67 (1.04)	9.79 (0.17)	3.25 (0.87)	8.14 (0.57)	0.50 (4.75)	7.18 (1.87)	2.75 (0.94)	1.09 (3.91)
**L_3**	2.78 (1.04)	9.66 (0.14)	2.25 (0.74)	8.83 (0.64)	1.12 (3.87)	6.52 (1.87)	2.60 (0.98)	0.73 (4.27)
**L_4**	2.59 (1.00)	9.59 (0.17)	1.86 (0.64)	9.37 (0.63)	0.65 (3.47)	6.62 (1.43)	2.56 (0.90)	1.60 (5.62)
**Bulk**	2.77 (1.07)	9.57 (0.15)	1.94 (0.61)	9.61 (0.62)	2.92 (3.53)	6.27 (1.43)	2.96 (0.97)	1.39 (5.74)

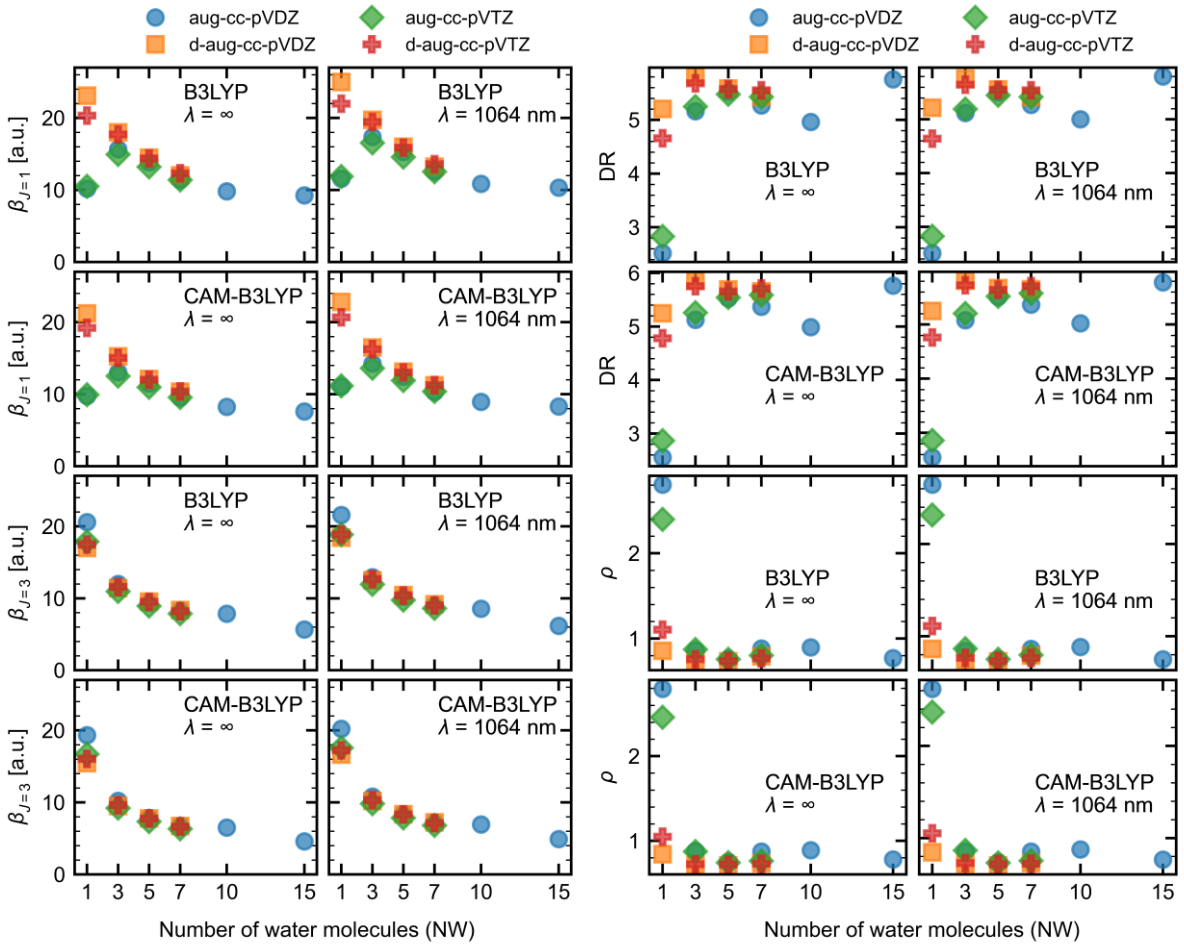

Figure S1. Evolution of $\beta_{J=1}$, $\beta_{J=3}$, DR, and ρ as a function of the number of water molecules (NW), as obtained at the TDDFT level using different XC functionals and atomic basis sets.

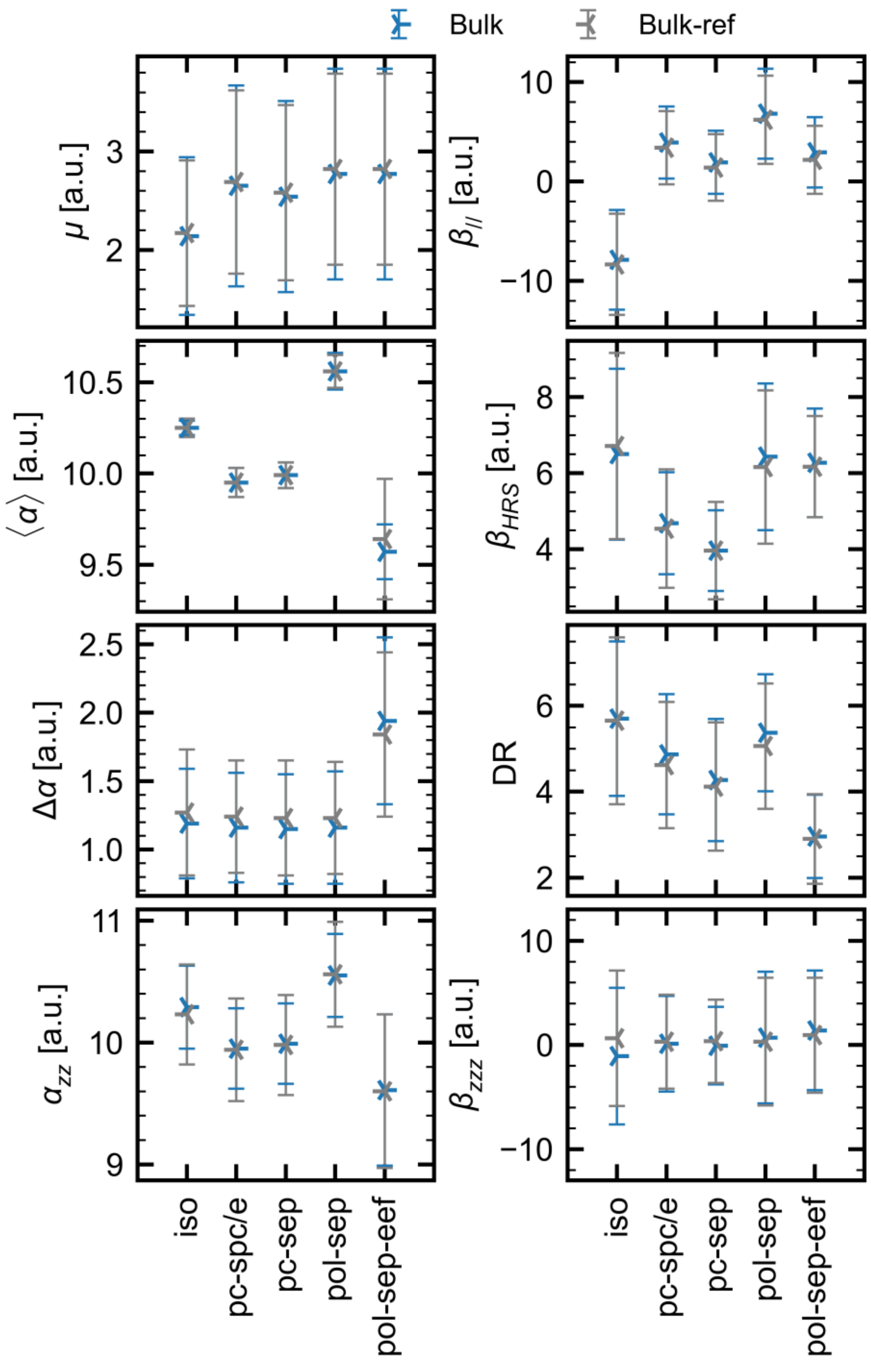

Figure S2. Comparison between the linear and nonlinear optical properties for two definitions of the bulk phase: the reference bulk, as obtained from MD simulations on the bulk, and the bulk-like region of the water slab, as calculated at the CAM-B3LYP/aug-cc-pVDZ level of approximation ($\lambda = 1064$ nm for the α and β quantities) for different embedding models. The μ, $\langle\alpha\rangle$, $\Delta\alpha$, α_{zz}, $\beta_{//}$, β_{HRS}, β_{zzz} (in a.u.) and DR averages are represented by the symbols whereas the standard deviations by error bars. All water molecules within a sphere of 15 Å radius were considered in the embedding approximation, except for **iso**, where no embedding was considered. The averages were performed over 100 snapshots extracted from the SPC/E trajectory.

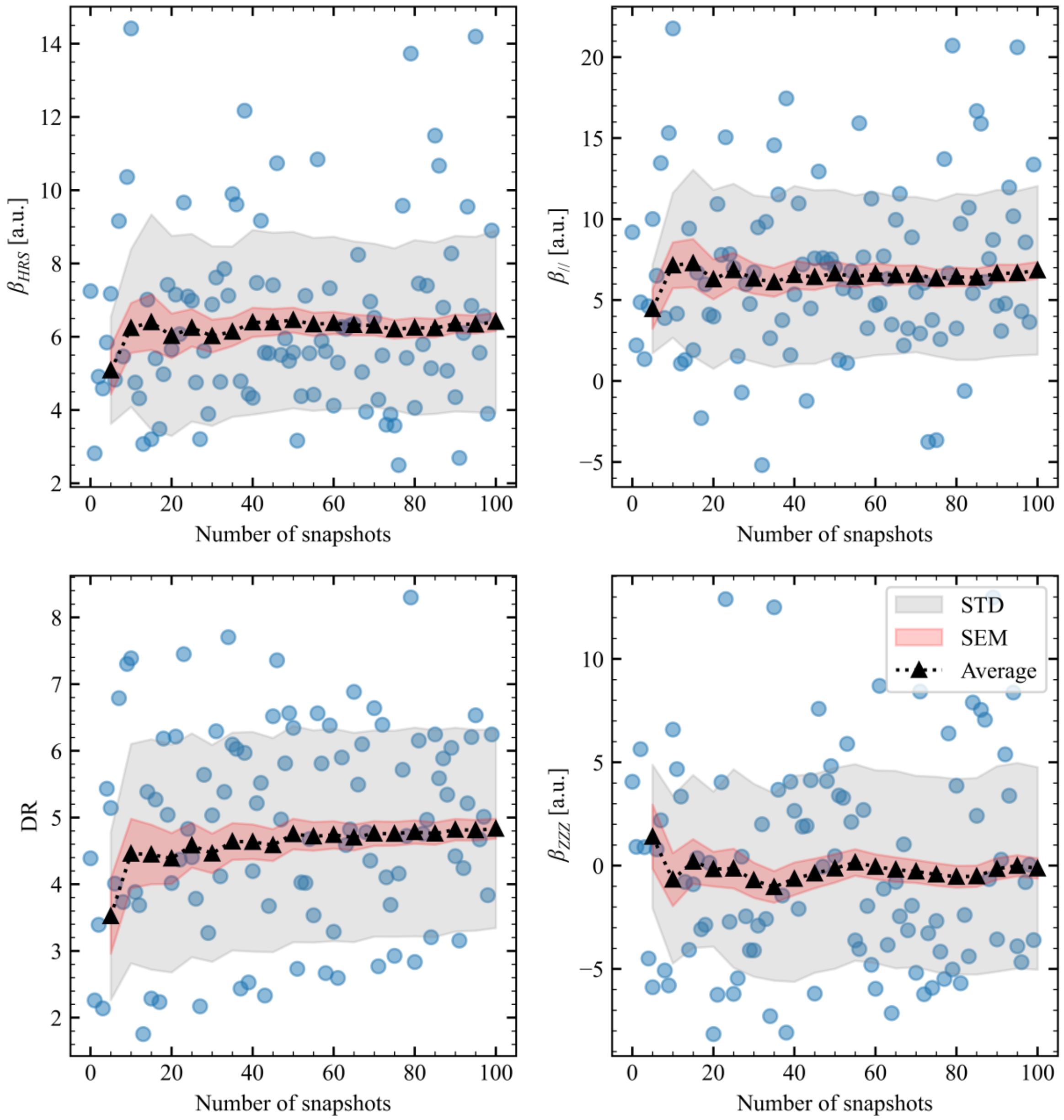

Figure S3. Cumulative average, standard deviation (STD), and standard error of the mean (SEM) of the nonlinear optical properties of the interface as a function of the number of snapshots. The results were obtained at the CAM-B3LYP/aug-cc-pVDZ level of approximation ($\lambda = 1064$ nm). All water molecules within a sphere of 15 Å radius were considered in the **pol-sep** surrounding approximation.

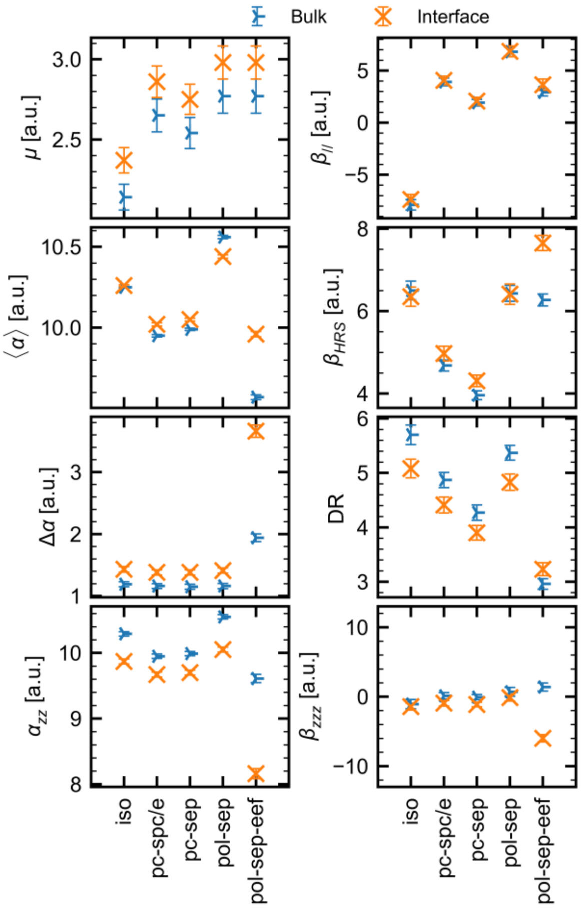

Figure S4. Comparison between the linear and nonlinear optical properties of the bulk and interface, as calculated at the CAM-B3LYP/aug-cc-pVDZ level of approximation ($\lambda = 1064$ nm for the α and β quantities) for different embedding models. The average values are represented by symbols whereas the standard errors of the mean are given by error bars. All water molecules within a sphere of 15 Å radius were considered in the embedding approximation, except for **iso**, where no embedding was considered. The averages were performed over 100 snapshots extracted from the SPC/E trajectory.

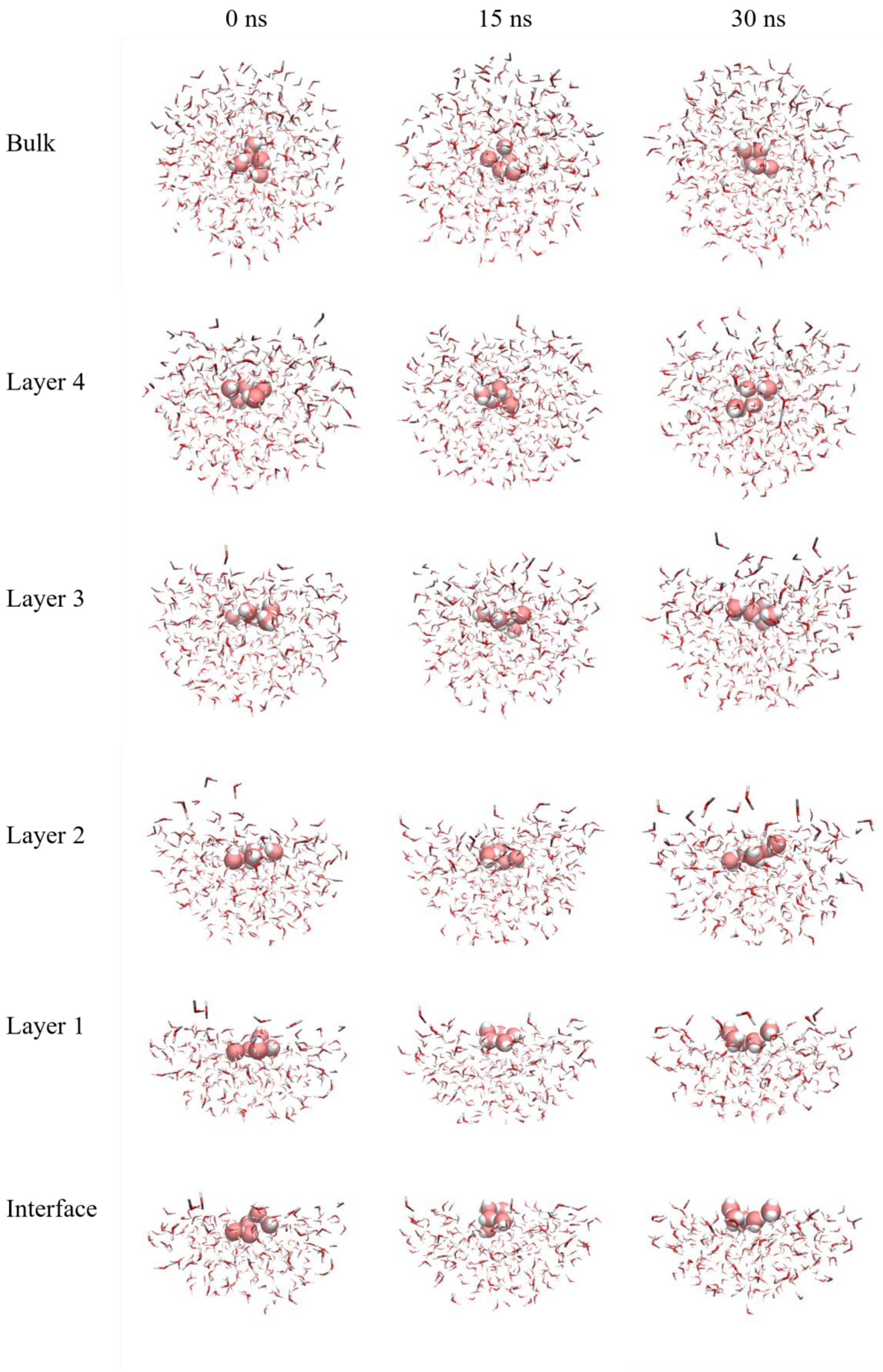

Figure S5. Extracted snapshots highlighting the QC core water molecules surrounded by the embedding water molecules. Note that the Interface configurations can include in the QC core water molecules from layers different from Layer 1, as observed, for instance, on the 15 ns snapshot.

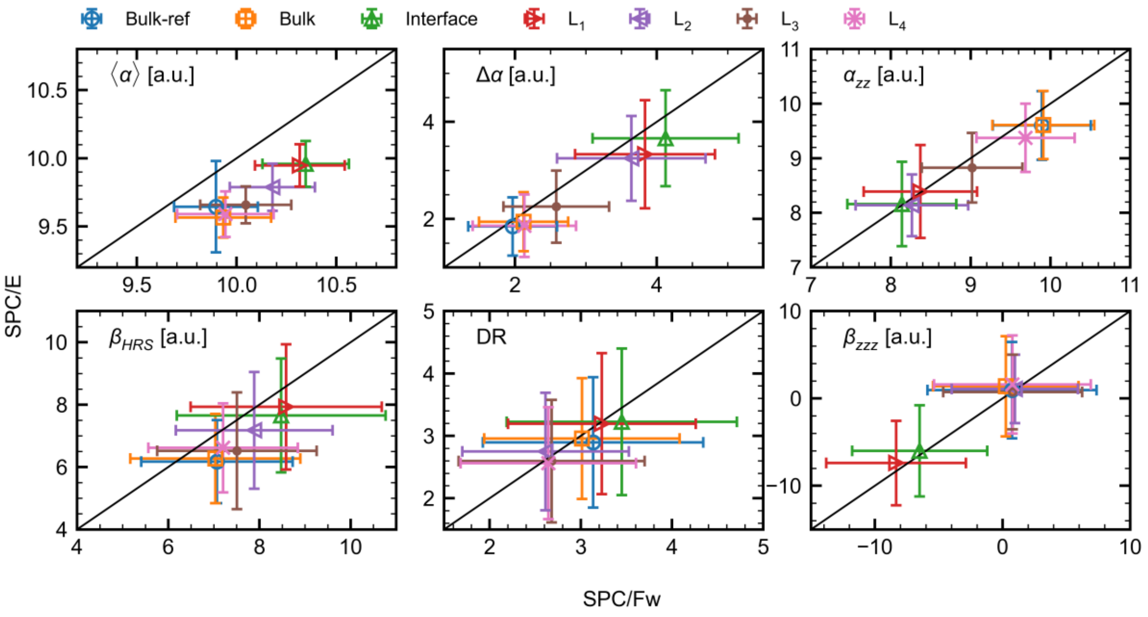

Figure S6. Relationships between the rigid (SPC/E) and flexible (SPC/Fw) water molecule force fields on the linear and nonlinear optical properties, as calculated at the CAM-B3LYP/aug-cc-pVDZ level of approximation ($\lambda = 1064$ nm for the α and β quantities). The average values are represented by symbols whereas the standard deviations by error bars. All water molecules within a sphere of 15 Å radius were considered in the **pol-sep-eef** approximation embedding. A set of 100 snapshots were extracted from the SPC/E and SCP/Fw trajectories.
